# CAR-T cell therapy in TP53-mutated CNS lymphoma: overcoming a high-risk genetic barrier

**DOI:** 10.3389/fmed.2025.1731589

**Published:** 2026-01-13

**Authors:** Danyang Li, Rui Liu, Zhonghua Fu, Fan Yang, Lixia Ma, Miaomiao Cao, Yuelu Guo, Biping Deng, Alex H. Chang, Qinlong Zheng, Xiaoyan Ke, Kai Hu

**Affiliations:** 1 Department of Lymphoma and Myeloma Research Center, Beijing GoBroad Hospital, Beijing, China; 2 Engineering Research Center of Gene Technology, Ministry of Education, Institute of Genetics, School of Life Sciences, Fudan University, Shanghai, China; 3 Shanghai YaKe Biotechnology Ltd., Shanghai, China; 4 Department of Medical Laboratory, Beijing GoBroad Boren Hospital, Beijing, China; 5 Department of Hematology, Peking University Third Hospital, Beijing, China

**Keywords:** CAR-T, cell therapy, CNSL, TME, TP53

## Abstract

**Background:**

Central nervous system lymphoma (CNSL) is a rare but aggressive subtype of lymphoma that presents significant therapeutic challenges. The prognosis for patients with CNSL varies significantly based on several genetic factors, including TP53 mutations, which are among the most critical determinants of treatment outcomes. Chimeric antigen receptor T (CAR-T) cell therapy has shown promising results in several hematological malignancies, including B-cell lymphomas. However, its efficacy in CNSL, particularly in patients with TP53 mutations, requires further investigation.

**Methods:**

A retrospective cohort study was conducted on 61 CNSL patients who had been treated at our institution from 2020 to 2024. The median follow-up time was 14.5 months. A total of 43 patients received CAR-T cell infusion therapy. The overall survival (OS) and progression-free survival (PFS) of patients harboring TP53 mutations (TP53+) and those with wild-type TP53 (TP53−) were compared. In addition, factors associated with patient prognosis were also identified.

**Results:**

Among the 43 patients who received CAR-T cell therapy, 17 harbored TP53 mutations. The median age of the cohort was 51.5 years, and 51.2% of the patients (22/43) were male. The overall response rate (ORR) and the complete response rate (CRR) in the TP53+ CAR-T+ group were both 64.5% (11/17), the median OS duration was 14.07 months (95% CI 12.63–∞), and the median PFS duration was 12.77 months (95% CI 6.33–∞). In the TP53-CAR-T+ group, the ORR was 73.3% (19/26), the CRR was 69.2% (18/26), the median OS duration was 33.47 months (95% CI 11.23–∞), and the median PFS duration was 22.4 months (95% CI 6.13–∞). In the subgroup analysis, the cell-of-origin (COO) classification was a key factor influencing the long-term survival of CSNL patients; in the TP53+ group, patients with non-germinal center B-cell-like (GCB) classification had longer OS compared to the GCB subtype (*p* = 0.003).

**Conclusion:**

CAR-T cell therapy is an effective treatment for CNSL patients harboring TP53 mutations and has the same efficacy as traditional treatment methods. Additionally, CAR-T cells may be more effective for TP53+ CSNL patients with a non-GCB classification.

## Introduction

Central nervous system (CNS) lymphoma includes primary CNS lymphoma (PCNSL) and secondary CNS involvement in systemic lymphoma (1). The first-line treatment for PCNSL is chemotherapy based on high-dose methotrexate (HD-MTX), with or without rituximab and radiotherapy (2). However, these conventional regimens are associated with severe side effects such as delayed neurotoxicity, which can lead to cognitive impairment. While there is no established standard treatment for PCNSL recurrence, temsirolimus, lenalidomide, temozolomide, and the Bruton’s tyrosine kinase (BTK) inhibitor ibrutinib are potential options for refractory patients. Over the past few decades, the prognosis of PCNSL patients has improved significantly (median OS: 26 months, 5-year survival rate: 31%) (3).

Histo-genetic and molecular analyses of PCNSL have identified ATM, TP53 (4), PTEN (5), PIK3CA (6), JAK3, CTNNB1, PTPN11, and KRAS as major determinants of pathogenesis, survival, and recurrence (7). TP53 alterations are established markers of poor prognosis in various cancers. P53 regulates apoptosis (8) and may also play a role in immune evasion and the induction of an immunosuppressive tumor microenvironment (TME) (9, 10), factors that can influence the cytotoxicity of chimeric antigen receptor (CAR)-T cells against large-cell B-cell lymphoma (LBCL). However, the impact of TP53 mutations on the efficacy of CAR-T cell therapy in CNSL patients remains unclear. In this observational study, we explored the influence of TP53 mutations on the efficacy of CAR-T cell therapy in CNSL patients.

## Methods

### Patients

**Figure 1:**
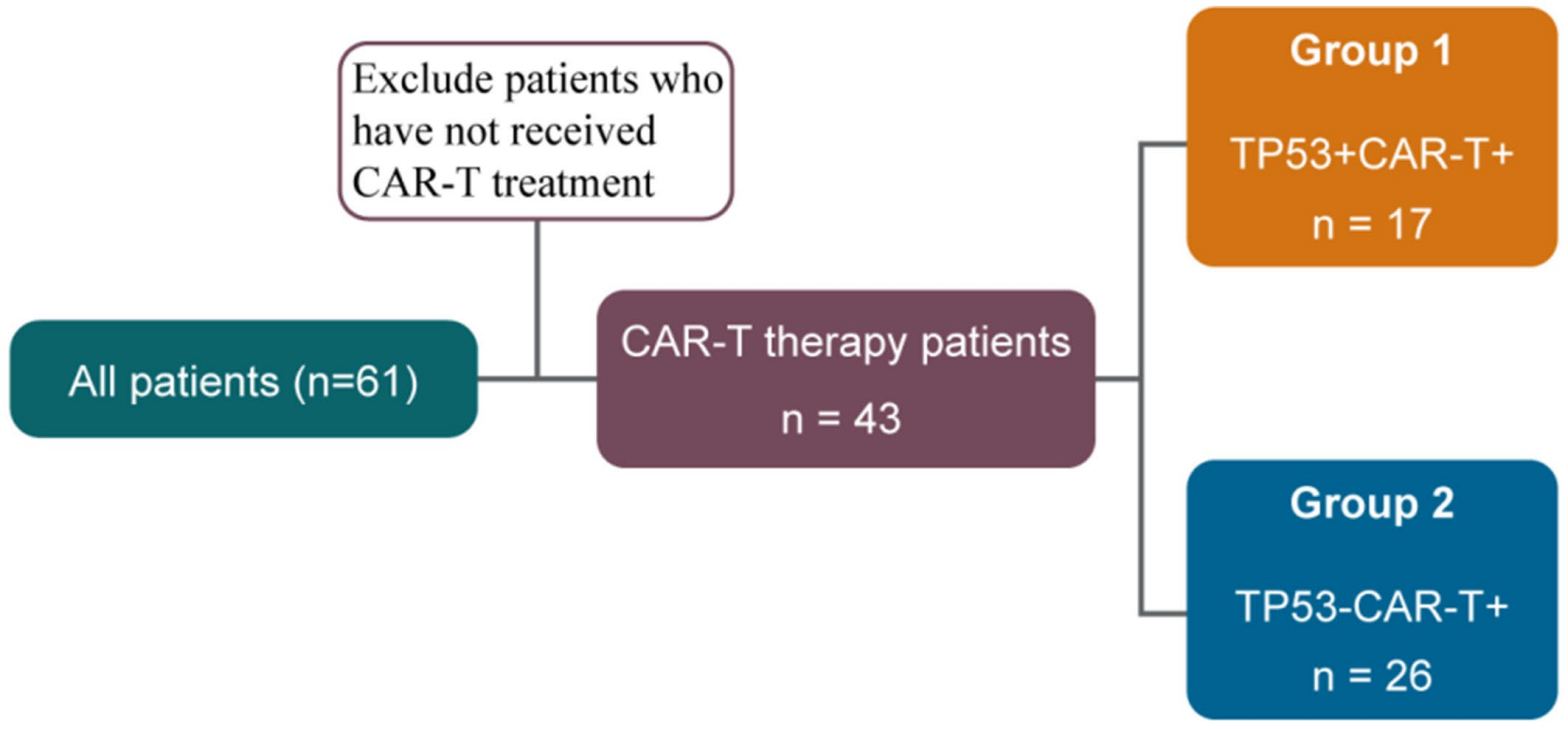
Flowchart of patient classification.

Sixty-one patients diagnosed with CNSL from 2020 to 2024 at Beijing GoBroad Hospital were enrolled. All patients were diagnosed with CSNL according to the current diagnostic criteria and had failed first-line treatment. According to the Lugano lymphoma efficacy evaluation criteria, treatment failure includes failure to achieve complete remission (CR)/partial remission (PR) after the first-line treatment, progression within 6 months after CR/PR, stable disease (SD) lasting for ≥6 months, or progressive disease (PD). Forty-three patients, including 22 men and 21 women, received CAR-T cell infusion therapy (designated as CAR-T+ in the study). The median age of the cohort was 51.5 years (range, 32–71 years). All patients who did not respond to first-line treatment or experienced a relapse received further treatment at our hospital. We conducted biopsies of the patients’ tumor masses and performed next-generation sequencing on the samples, and TP53 mutations were detected. As shown in Figure 1, 17 CAR-T+ patients harbored TP53 mutations (TP53+ CAR-T+), whereas the remaining 26 patients were negative for TP53 mutations (TP53-CAR-T+).

The baseline clinical characteristics of the patients are listed in Table 1. Survival time was defined as the period from the infusion of CAR-T cell therapy to the last follow-up, relapse, or death. This study was approved by the Ethics Committee of Beijing GoBroad Hospital. All patients participated in the clinical trial using the CAR-T cell product (11) from Shanghai YaKe Biotechnology Co., Ltd., in China (clinical registration number ChiCTR2100055062). Written informed consent was obtained from all participants or their families prior to obtaining samples.

**Table 1:** Patients’ characteristics.

Characteristics	Whole cohort		TP53 mutation (*n* = 17)		TP53 WT (*n* = 26)		
	No.	%	No.	%	No.	%	*p*-value^a^
	43	100	17	39.5	26	60.5	
Age							
Median (range)	51.5 (32–71)		51 (32–70)		52 (32–71)		
≥60 years, *n* (%)	12	27.91	7	41.18	5	19.23	0.859
Sex							0.289
Female, *n* (%)	21	48.84	11	64.71	10	38.46	
Male, *n* (%)	22	51.16	15	88.24	7	26.92	
Diagnosis							0.722
PCNSL	29	67.44	12	70.59	17	65.38	
SCNSL	14	32.56	5	29.41	9	34.62	
Previous treatment lines							0.021
≥3	22	51.16	5	29.41	17	65.38	
<3	21	48.84	12	70.59	9	34.62	
ECOG score							0.502
≥3	8	18.60	4	23.53	4	15.38	
<3	35	81.40	13	76.47	22	84.62	
COO							0.055
GCB	25	58.14	10	58.82	15	57.69	
Non-GCB	9	20.93	6	35.29	3	11.54	
Unknown	9	20.93	1	5.88	8	30.77	
IPI score							0.973
≥3	10	23.26	4	23.53	6	23.08	
<3	33	76.74	13	76.47	20	76.92	
MYC rearrangement							0.851
Yes	8	18.60	3	17.65	5	19.23	
No	18	41.86	8	47.06	10	38.46	
Unknown	17	39.53	6	35.29	11	42.31	
BCL-2 rearrangement							0.851
Yes	7	16.28	3	17.65	4	15.38	
No	16	37.21	7	41.18	9	34.62	
Unknown	20	46.51	7	41.18	13	50.00	
BCL-6 rearrangement							0.622
Yes	10	23.26	3	17.65	7	26.92	
No	12	27.91	6	35.29	6	23.08	
Unknown	21	48.84	8	47.06	13	50.00	
Pre-treatment efficacy of CAR-T therapy							0.464
CR	21	48.84	7	41.18	14	53.85	
PR	9	20.93	3	17.65	6	23.08	
SD	7	16.28	1	5.88	0	0.00	
PD	12	27.91	6	35.29	6	23.08	
The site of tumor invasion							0.255
Brain parenchyma	23	53.49	9	52.94	14	53.85	
Secondary brain parenchyma	5	11.63	1	5.88	4	15.38	
Secondary cerebrospinal fluid	2	4.65	0	0.00	2	7.69	
Cerebrospinal fluid and brain parenchyma	11	25.58	5	29.41	6	23.08	
Secondary spinal canal	2	4.65	2	11.76	0	0.00	

COO, cell of origin; GCB, germinal center B cell; Pearson’s chi-squared test.

### CD19 CAR-T product

A lentiviral vector encoding a CD19 CAR with a 4-1BB costimulatory domain and a CD3-zeta signaling domain was constructed. The CD19 recognition domain consisted of a single-chain fragment variable region derived from the FMC63 monoclonal antibody. This CD19 CAR-T cell was available as an investigational new drug (IND) product from Shanghai YaKe Biotechnology Co., Ltd.

### Endpoints

The primary endpoints were overall response rate (ORR), complete response rate (CRR), overall survival (OS), progression-free survival (PFS), and adverse events. OS was measured from the date of CAR-T cell therapy to the date of death or the last follow-up. PFS was measured from the initiation of CAR-T therapy to the date of disease progression or death due to the disease. Treatment efficacy was assessed according to the Lugano 2014 criteria. The exploratory endpoint was to evaluate the impact of TP53 mutation status and other factors on clinical outcomes.

### Statistical analysis

The demographic and other baseline data have been presented as frequencies and percentages. The probabilities of OS and PFS were calculated using the Kaplan–Meier method and compared using the log-rank test. The 95% CI for survival was calculated using GraphPad Prism V.9.0 software. SPSS version 26.0 and GraphPad Prism version 9.0 were used for data analysis. A two-sided *p*-value of <0.05 was considered statistically significant.

## Results

### CAR-T therapy is effective in patients with TP53 mutations

Of the 43 CNSL patients included in the study, 17 harbored TP53 mutations. The median age was 51.5 years, and 22 patients (51.2%) were male. Table 2 provides a detailed description of the TP53 mutation types. Before the CAR-T treatment, the tumor burden of the patients and the efficacy of the before CAR-T treatment were evaluated. Among TP53− patients, 18 (69.2%) achieved complete response (CR), and 10 patients (38.5%) underwent CAR-T treatment with tumor mass. Among TP53+ patients, 7 (41.2%) achieved CR, and 9 (52.9%) underwent CAR-T treatment with tumor mass (Table 3).

**Table 2:** The type of TP53 mutation in patients.

Patients No.	cDNA level	Protein level	Patients No.	cDNA level	Protein level
1	c.650T>G	p.V217G	10	c.215C>G	p.Pro72Arg
2	c.604C>T	p.R202C	11	c.916C>T/c.743G>A	—
3	c.437G>A	p.W146X	12	—	—
4	c.391A>T	p.N131Y	13	c.742C>G/c.641A>G	p.R248G/p.H214R
5	c.733G>A	p.G245S	14	c.404G>A	p.C135Y
6	c.408A>C	p.Q136H	15	—	—
7	—	—	16	c.215C>G	p.Pro72Arg
8	c.535C>T	p.H179Y	17	c.524G>A	p.Arg175His
9	c.581T>G	p.L194R			

Data of patient 7, patient 12, and patient 15 were lost because they were obtained through a next-generation sequencing conducted outside the hospital.

**Table 3:** Tumor burden and efficacy before CAR-T therapy.

Characteristics	TP53 mutation (*n* = 17)	TP53 WT (*n* = 26)	*p*-value
	No. (%)	No. (%)	
Tumor burden before CAR-T			0.35
Yes	9 (52.9)	10 (38.5)	
No	8 (47.1)	16 (61.5)	
Efficacy before CAR-T			0.119
CR	7 (41.2)	18 (69.2)	
PR	4 (23.5)	5 (19.2)	
PD	6 (35.3)	3 (11.5)	

**Figure 2:**
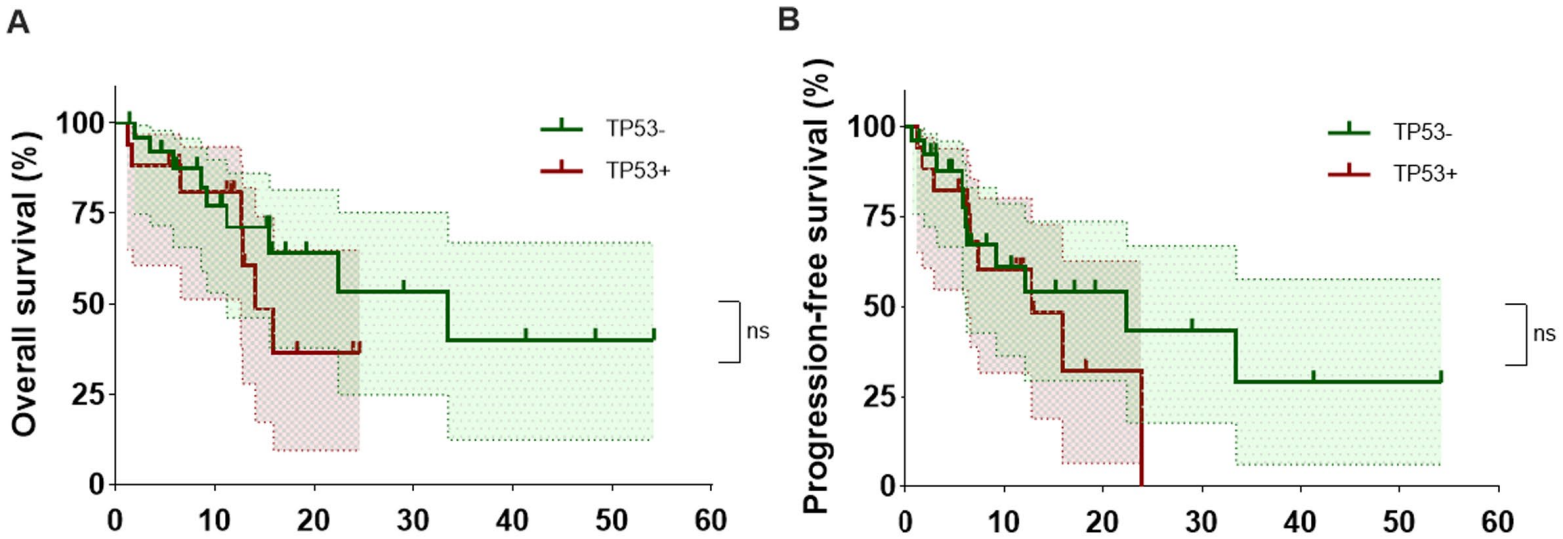
Impact of TP53 mutation status on the OS and PFS duration after CAR-T cell therapy. **(A)** The OS of TP53-mutated (TP53+) and TP53-wild type (TP53−) patients. **(B)** The PFS of TP53+ and TP53− patients. NS, no significance.

The ORR and CRR of TP53+ CAR-T+ patients were both 64.5% (11/17). The median OS duration was 14.07 months (95% CI 12.63–∞), and the median PFS was 12.77 months (95% CI 6.33–∞). In the TP53-CAR-T+ group, the ORR was 73.3% (19/26), the CRR was 69.2% (18/26), the median OS duration was 33.47 months (95% CI 11.23–∞), and the median PFS duration was 22.4 months (95% CI 6.13–∞) (Figure 2 and Table 4). Compared with patients with TP53 mutations, those with wild-type TP53 had longer OS and PFS after CAR-T cell therapy. However, there was no statistically significant difference between the two groups of patients. Although it is unclear whether TP53 affects the prognosis of patients withe central nervous lymphoma, our research suggest that CAR-T therapy also effective in CNSL patients with TP53 mutations (12, 13).

**Table 4:** Efficacy of CAR-T cell therapy in TP53+ and TP53− patients.

*n* (95% CI)	CAR-T therapy (*n* = 43)			
	ORR	CRR	OS	PFS
TP53+ (*n* = 17)	11/17 (64.5%)	11/17 (64.5%)	14.07 (12.63–∞)	12.77 (6.33–∞)
TP53− (*n* = 26)	19/26 (73.3%)	18/26 (69.2%)	33.47 (11.23–∞)	22.4 (6.13–∞)

ORR, overall response rate; CRR, complete response rate; OS, overall survival; PFS, progressive-free survival; CR, complete response; PR, partial response; SD, stable disease; PD, progressive disease; TP53+, presence of TP53 mutation; TP53−, TP53 wild-type. ∞, upper bound not estimable, due to the insufficient follow-up time for some patients, it is impossible to estimate the upper limit of the confidence interval.

### Adverse events

Among the TP53+ CAR-T+ patients, 41.2% (7/17) did not experience cytokine release syndrome (CRS), 52.9% (9/17) experienced grade 1–2 CRS, and only 1 (5.9%) experienced grade 3–4 CRS. The reactions were controllable for all patients. Furthermore, one patient had grade 3–4 immune effector cell-associated neurotoxicity syndrome (ICANS). In the TP53-CAR-T+ group, 46.2% of the patients (12/26) did not experience CRS, 46.2% (12/26) experienced grade 1–2 CRS, and only 7.7% (2/26) experienced grade 3–4 CRS. Two patients had grade 3–4 ICANS (Table 5). Based on these results, no conclusion could be drawn regarding the impact of TP53 mutations on the safety of CAR-T cell therapy. No other hematological-related toxicities were observed.

**Table 5:** Adverse events in TP53+ and TP53− patients after CAR-T cell therapy.

Adverse events	CRS			ICANS		
	Grade 0	Grades 1–2	Grades 3–4	Grade 0	Grades 1–2	Grades 3–4
Group 1	7/17 (41.2%)	9/17 (52.9%)	1/17 (5.9%)	15/17 (88.2%)	1/17 (5.9%)	1/17 (5.9%)
Group 2	12/26 (46.2%)	12/26 (46.2%)	2/26 (7.7%)	24/26 (92.3%)	0	2 (7.7%)
Total	19/43 (44.2%)	21/43 (48.8%)	3/43 (7.0%)	39/43 (90.7%)	17/43 (39.5%)	3/43 (7.0%)

Group 1, TP53 mutation with CAR-T therapy; Group 2, TP53 no mutation with CAR-T therapy.

### Subgroup analysis

**Figure 3:**
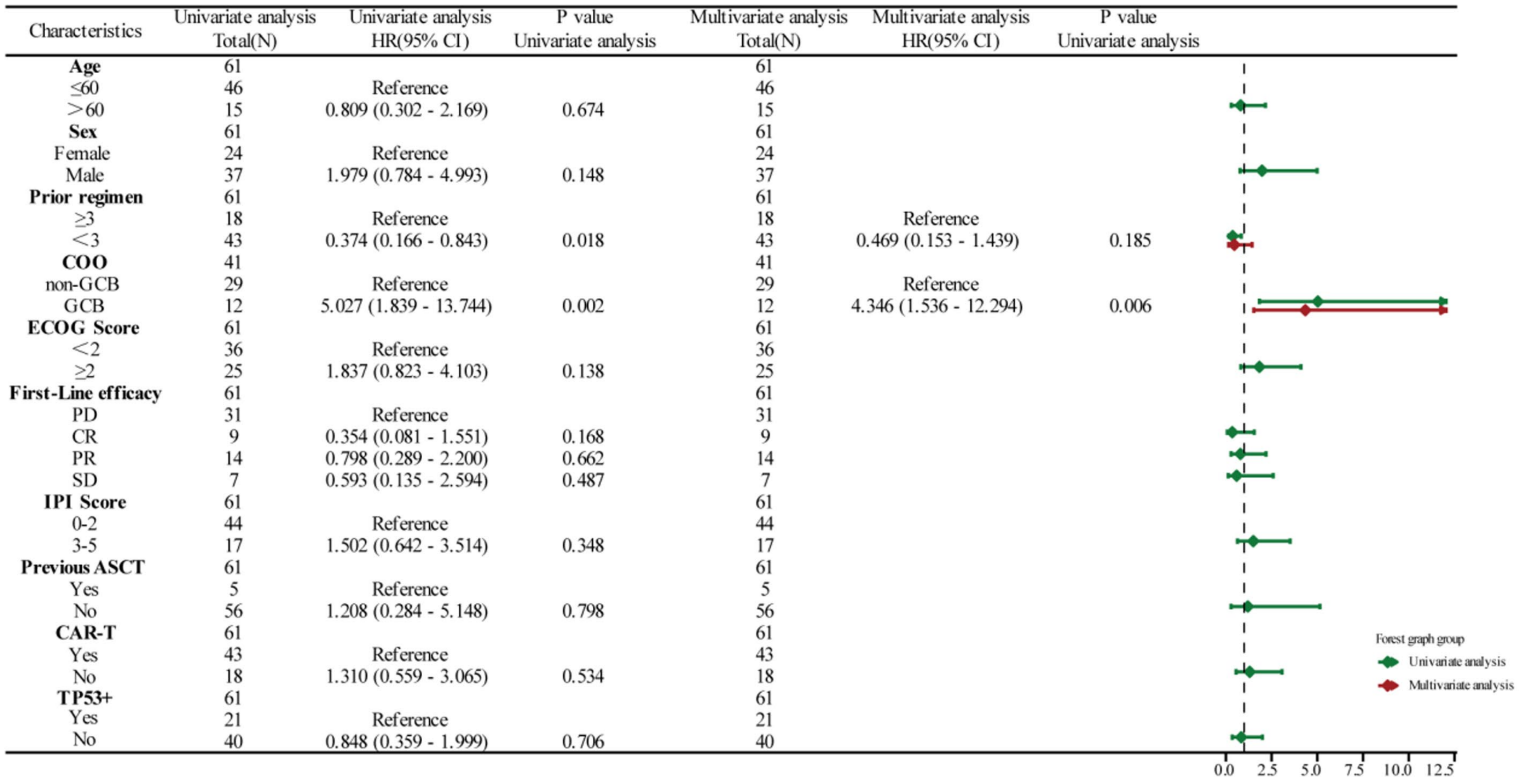
Univariate/multivariate COX regression analysis for all 61 patients in OS.

**Figure 4:**
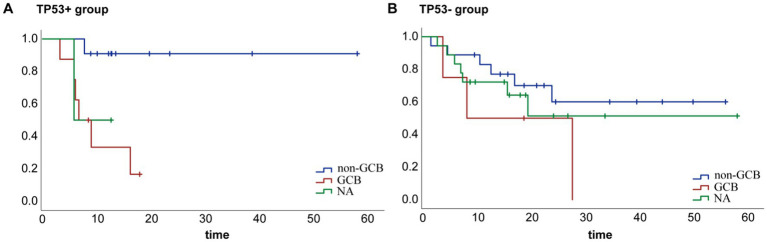
Impact of COO classification on patient prognosis. **(A)** Non-GCB patients had a better prognosis compared to GCB patients in the TP53+ group. **(B)** There was no significant difference in the OS of the subgroups in the TP53− group.

The impact of patient age, sex, prior treatment regimen, Eastern Cooperative Oncology Group score, first-line efficacy, International Prognostic Index score, and previous ASCT was analyzed by univariate and multivariate Cox regression analyses. All 61 patients were included in the analyses. We identified cell-of-origin (COO) as a key factor influencing the OS (Figure 3). TP53+ patients with the non-germinal center B-cell-like (GCB) subtype had longer survival compared to patients with the GCB lymphoma subtype. In contrast, no significant difference was observed in the prognosis of the GCB and non-GCB subgroups in patients with wild-type TP53 (Figure 4). Interestingly, patients with TP53 mutations exhibited shorter OS after conventional therapies (such as chemotherapy and stem cell transplantation) when compared to those without TP53 mutations, suggesting that the mutation may confer resistance to standard treatments.

## Discussion

The results of this study indicate that CAR-T therapy is effective for CNSL patients with TP53 mutations. While significant ORR and CRR were observed in the TP53+ CAR-T+ group, the median OS and median PFS were longer in the TP53-CAR-T+ group. Nevertheless, the presence of TP53 mutations did not significantly impact the PFS in patients treated with CAR-T cells. In addition, the frequency of adverse events, particularly CRS, was also similar in the TP53+ and TP53− groups. We also identified COO classification as a key factor influencing patient survival. Furthermore, patients harboring TP53 mutations had shorter OS after conventional therapies, which suggests that TP53 mutations may confer resistance to standard treatments.

Although TP53 mutations are not established prognostic factors for CNSL, they have been reported in patients with CNSL (12, 13). In a prospective study on patients with diffuse large B-cell lymphoma (DLBCL), mutations in the MYD88 and TP53 genes were identified as sensitive, specific, and accurate predictors of overall mortality and disease progression (14). In the majority of patients with anaplastic lymphoma kinase (ALK)-positive anaplastic large-cell lymphoma (ALCL), long-term survival can be achieved with CHOP chemotherapy. However, TP53 deletion is a risk factor in ALK+ALCL patients treated with the CHOP-based regimens (15). A large retrospective study of patients with refractory/relapsed aggressive B-cell non-Hodgkin’s lymphoma (r/r B-NHL) reported that CAR-T cell therapy achieved an ORR of 87.1% and a CRR of 45.2%. The median PFS was 14.8 months for patients harboring TP53 mutations, and the 24-month OS was 56.3% (16), indicating that CAR-T cell therapy is effective regardless of TP53 mutations. Du et al. (17) constructed a TP53 missense mutation-based risk model for DLBCL using bioinformatics analysis and machine learning, and confirmed that patients with TP53 mutations had a worse prognosis. In addition, multiple studies have shown that CAR-T cell therapy is effective in non-Hodgkin lymphoma patients with TP53 mutations (18–21).

Although our findings provide constructive guidance on the efficacy of CAR-T therapy for CNSL with TP53 mutations, there are still some limitations to our study. First, this study was conducted at a single center, which may limit the general applicability of the results. Second, the sample size was relatively small, and only 17 patients had TP53 mutations, which may lead to insufficient statistical power and make it difficult to draw highly representative conclusions. Therefore, future multi-center and large-sample studies are needed to enhance the reliability and validity of the results. Nevertheless, this study provides a basis for the future treatment of CNSL, such as the use of combination therapy (22), targeted treatments (23, 24), and new delivery systems such as nanomaterial delivery systems (25–27).

## Conclusion

Our study indicated that CAR-T cell therapy is an effective treatment for CNSL with TP53 mutations and has comparable efficacy to traditional treatment methods in patients without TP53 mutations. However, due to relatively lower efficacy in TP53+ patients, it may be necessary to adopt alternative or combination therapies. Apart from the TP53 mutation status, the COO classification should also be considered when choosing the most appropriate treatment plan for CNSL. CAR-T cell therapy may have better efficacy in patients with TP53 mutations and non-GCB classification.

## Data Availability

The raw data supporting the conclusions of this article will be made available by the authors, without undue reservation.
